# Truffles

**Published:** 1881-07

**Authors:** 


### TRUFFLES.

The truffle is described as a mysterious vegetable which does not give the least sign of vegetation, either in the ground or out of it.

The truffle is found under three species of trees only—the filbert, and the white and the red oak (the last two in particular), and of late farmers and others of the county of Perigord have found it profitable to sow the white and red oak acorn for the only object of raising truffles. The young plants will produce truffles the fifth or sixth year, and often the crop of the tenth year is sufficient to remunerate the farmer for all the labor and interest invested.

The finding of the truffles would be due to mere luck or hard labor if it were not for the keen scent of the sows, which, in that strange hunt, are trained to take the place of the faithful pointer dog, some arriving to such a degree of education that they bring very high prices. Sows trained for hunting truffles are fed entirely on acorns—never anything else—and, during the season, once a day only, after the day's work is over.

Truffle hunting is a specialty, and the men devoted to it depend on the short season of 40 to 50 days to earn enough to take care of themselves and their sows the rest of the year. The hunter cannot employ his time at anything else. He has all he can do to gather, every day, the necessary supply of acorns needed by his useful animal. Hunters generally start the day after Christmas. The whole outfit consists of the sow, fastened by a hind leg; one bag containing bread and cheese for the man and acorns for the sow; another bag ready to receive the truffles; a blanket and a cane. The poor brute being led away seems delighted, well knowing that it will be paid for every truffle it finds.

They are no sooner in the woods than the sow is let loose and begins to hunt, the man keeping close watch behind. The sow will go slowly over the ground and never root until it scents the precious vegetable. Then the skill of the hunter is required, for he must be ready to strike the sow on the snout the moment the truffle is in sight. Her sowship will retire with a groan. The man then picks up and bags the truffles, and gives the poor animal one or two acorns for its trouble. Man and sow stay out in the woods until driven away by a hard storm or by want of provisions, in which case they stop at the nearest farmhouse and deposit the result of their labor so far. Having rested and gathered the necessary acorns, they start again, and keep doing the same until the tuber has disappeared from the ground in the same mysterious manner as it came.

The preparation of the article is very simple. It is carefully washed and brushed. Some are peeled ; others are left in their natural state ; all are boiled just once, then put in tin cans, adding a wineglass of white wine to keep them moist. The cans, after being hermetically sealed, are submitted to a second boiling to insure preservation. Truffles are then ready to be shipped to any climate, and appear on the tables of gourmands the world over.—*Pacific Rural Press.*

